# Case of Lumbar Abscess

**Published:** 1802-07

**Authors:** J. Wilson

**Affiliations:** Gillingham, in Kent


### *Case of Lumbar Abscess*; *communicated by Mr.* J. Wilson, *of Gillingham, in Kent.*

To the Editors of the Medical and Physical Journal.

Gentlemen,

As the beſt means of making a very intereſting, and indeed a very doubtful caſe public, I beg leave to requeſt that you will inſert in your valuable Journal the following relation of a caſe which has occurred here, and which it is preſumed may not be unacceptable to a large body of your readers.

Mrs. Hazlewood, about thirty-five years of age, was brought from London to Gillingham, and received into the Poor-Houſe, on account of a tumour in the abdomen on the right ſide, and a ſwelling of the thigh and leg. She had been diſcharged from Guy's Hoſpital as incurable, and had been an out patient of the Surry Diſpenſary, without receiving any alleviation of her ſufferings. The gentleman who attended the Poor-Houſe (Mr. Congreve of Brompton) conſidering the caſe obſcure, and preſenting ſome ſymptoms worthy of remark, did me the favor to requeſt my attendance with him. The tumour in the abdomen, which had been rapidly increaſing, was then about the ſize of the hand when clenched; it evidently contained a fluid, and was ſo circumſcribed in its external form as to leave but little doubt as to its being contained in a ſac or cyſt. It was ſituated above the tendon of the oblique muſcle, ſtretching from thence obliquely upwards, and towards the ilium; it was not diſcoloured. The thigh, which was bent towards the abdomen, was much enlarged; the leg alſo was ſwelled, very painful, and the knee bent. The tumour was not ſo exquiſitely tender, nor did ſhe at any time ſuffer ſo much pain there as in the extremity. She complained of pain in the loins; ſhe could not paſs urine except when on her hands and knees, and then in ſmall quantity and much diſtreſs. She had not any inteſtinal evacuation but when an enema was adminiſtered; ſhe had not menſtruated ſince the commencement of her complaint. Her breaſts were abſolutely gone; her pulſe was ſmall and quick; ſhe was much emaciated; had the hippocratic countenance; and without the aid of opiates her nights were ſleepleſs. She had borne ſeveral children. Judging from the ſite and form of the tumour, with other concurring appearances, I hazarded an opinion that it was an ovarian dropſy. Mr. Congreve was diſpoſed to conſider it a ventral hernia. About a week after, on the annual change of pariſh officers taking place, place, I was nominated to the ſuperintendance of the poor, and felt myſelf happy in the opportunity afforded me of inveſtigating this caſe by a ſcrutinizing inquiry. When I deſired a particular hiſtory of her diſeaſe, ſhe gave me the following information. Nearly four months before the ſwelling in the thigh and leg came on, which was accompanied by ſo much pain, that ſhe was compelled to apply for medical aid. The thigh had been bliſtered and ſome other means uſed, but her miſery continued daily increaſing. A ſhort time after the thigh and leg began to enlarge, ſhe felt a hard body, nearly the ſize of a large marble, in the ſame ſide of the abdomen, but this was ſoon loſt in the augmenting bulk and fluctuation of the abdominal tumour. A difficulty in evacuating the contents of the bladder and rectum now ſupervened, and the ſwelling of the leg and thigh, with proportionate pain, was gradually increaſing. Her ſiſter, who came from London to ſee her a few days before her deceaſe, gave me farther intelligence, but I ſhall relate it in the order of time in which it was received, and leave it to the reader to connect it with the above. Such was her ſtate when ſhe came under my care. I invited ſeveral medical gentlemen to examine the caſe, and amongſt others, Dr. Vaughan of Rocheſter, whoſe talents are too well known both here and in the medical circle of the metropolis to make any encomiums from me neceſſary; he thought it involved in much obſcurity, but recommended the ung. hydrargyri to be rubbed upon the tumour and a bandage applied, with a view to promote abſorption. This treatment was not perſiſted in, becauſe the patient became extremely anxious to obtain more ſpeedy relief than could be hoped for from ſuch proceſs, however rational. I mentioned to the Doctor my opinion of its being an ovarian dropſy, and thought that opinion ſtrengthened by the circumſtance of her having loſt her breaſts, and ceaſed to menſtruate about the beginning of her complaint; but he did not coincide with me in idea that the ovaria were ſtrictly neceſſary to menſtruation, and moreover, urged that the left ovarium offered no appearance of diſeaſe. The ſymptoms becoming more urgent, the patient was examined, per vaginam, to diſcover whether the uterus was affected. On introducing the finger within the vagina a protruſion was plainly felt on the ſide next to the tumour, and evidently diminiſhing its paſſage. The diſtance being ſo great between the external and internal points of contact, I could not feel any fluctuation internally, when I applied my other hand to the abdomen and preſſed the tumour down, though I felt the whole of the protruding part in the vagina enlarge. I did not then feel the uterus. On the 30th of April I reſolved on the operation as the only poſſible means to ſave or protract her her life; partly, becaufe the tumour began to point exter-nally, which made me fear it would break; and partly to comply with the poor creature's wifh, that fomething fhould be done to procure her eafe. Before I proceeded, I wifhed to examine accurately the ftate and fituation of the uterus, and on introducing the index finger as far as I could, I felt the os uteri very plainly, lying high, and receding above the vaginal protrufion. Mr. Brenan, (a furgeon in the navy, and whofe opinion refpecting the cafe accorded with my own) alfo being prefent, examined, and felt the uterus diftinctly. I was the more particular on this point, becaufe the derangement confequent to fo large a tumour might have been embarraffing, and any miftake dangerous.

The patient being placed on her back with the pofteriors over the edge of the bed, and the thighs fupported and feparated by af-fiftants, I took a fmall trocar, and guarding the points with my fore finger, introduced it into the vagina. When the finger came in contact with the tumour I plunged the trocar in, penetrating about an inch, in a direction obliquely upwards, and, as near as I can guefs, parallel to a line drawn from the pubes to the fuperior fpinous procefs of the ilium; on withdrawing the trocar, and leaving the cannula within the orifice, about three pints of a fluid, fcarcely turbid, paffed off, and towards the latter part fomething like pus, with fmall floccules refembling the coagulated lymph often found in the abdomen, thorax, &c. The external tumour fubfided as the fluid drained off; the cannula was left in the orifice, and a bandage paffed round the abdomen. She was ordered to lie with her body raifed, and turned towards her left fide. As foon as fhe was laid on the bed, fhe made water with great eafe. In the evening fhe com-plained of prickling pains refembling the fenfations after a limb has been what is termed afleep, but more intenfe. Nearly a pint more difcharged; no pus, but a portion of membrane re-fembling part of a cyft or fac.

May 1. Had a very bad night; no ftool; made water eafily; very low; prickling pain in the abdomen; little difcharge; upper part of the tumour hard and unequal like fchirrhus; thought there might be fcirrhous adhefion to the rectum.

2d. Evacuated nearly three pints, fimilar to what paffed when tapped; no ftool; ordered an enema, which brought away a fmall quantity of fæces like the grounds of coffee, and very fœtid; the tumour again enlarging.

3d. Difcharged nearly three pints; tumour ftill enlarging; withdrew the cannula becaufe it irritated the vagina.

4th. Paffed about a pint, very fœtid, refembling the dif-charge per anum in colour. The right labium pudendi enlarged; larged; ſuggeſted the probability of a communication between the tumour and the rectum; more convinced of there having been at firſt either a ſac or cyſt, preſuming, that when the cannula was withdrawn the orifice in the cyſt had receded from the line of the puncture in the vagina, and the fluid had conſequently eſcaped into the cellular membrane.

5th. Diſcharge and other ſymptoms as yeſterday.

6th. About half a pint with much pus, very dark coloured; an enema produced a very ſparing evacuation.

7th. Rigor at eleven, a. m. and at four, p. m. diſcharge as yeſterday; no ſtool; makes water very well; tumour at a ſtand.

I now learned from the ſiſter, who came to ſee her, that ſix months ago ſhe had a fever, after which ſhe did not menſtruate, and the whole abdomen became enlarged; but on lifting too heavy a weight ſhe brought on an exceſſive flow of menſes for a fortnight; and immediately ſucceeding that time her thigh and leg began to ſwell. She had, as I obſerved before, always complained of a pain in her loins.

8th. Rigor and diſcharge as yeſterday; the ſurface of the tumour threatens ſphacelus.

9th. The tumour becoming of a leaden hue, and bliſtered; three rigors; ſinking; at twelve, p. m. an hæmorrhage from the vagina ſupervened, which carried her off in a few minutes.

Permiſſion being obtained to open the body, it was done in the preſence of Mr. Congreve and a neighbouring practitioner. On making the inciſion through the abdominal muſcles, the firſt object that attracted our notice was a large coagulum of blood about the ſize of a gooſe's egg, occupying that part of the tumour which was ſphacelated. The cavity of the tumour contained a pint of pus mixed with coagulated blood: it reached from the perinæum along the ſide of the vagina and rectum, which were protected by a condenſation of the cellular membrane, forming the parietes of the tumour next thoſe parts. On the other ſide it paſſed along the ilium to the lumbar region; the back part being the pſoa muſcles, which were almoſt ſuppurated away, and leaving the internal iliac artery bare through its whole length. The iſchium, from the ſpinous proceſs upwards, the ſacrum and the lumbar vertebræ, where ſome pus was lodged, were all carious. The cervix veſicæ was ſlightly inflamed on the left ſide, where it had been preſſed againſt the pubes by the tumour before it was tapped. The uterus, ovaria, and Fallopian tubes, the vagina and rectum, bore not the leaſt appearance of diſeaſe; nor were there any other marks of diſeaſe to be met with. We did not diſcover the veſſel that had produced the hæmorrhage. I ſhould have mentioned, in another place, that the ſwelling of the leg and thigh was was nearly fubfided, but that fhe complained of pain in the leg and foot to the laft. As to the former, there can be no difficulty in fuppofing it caufed by lymph obftructed by the afcending lymphatics ; nor the exquifite pain as caufed by preffure or difeafe in the ifchiadic nerve.

This, Gentlemen, being a faithful detail of the fymptoms when the patient was alive, and of the appearances on the infpection of the body when dead, we muft, I think, on a review of the whole, be compelled to acknowledge the cafe either a lumbar abfcefs, complicated with an encyfted tumour, or a lumber abfcefs itfelf, under an uncommon form. What the event of an earlier operation might have been we can only furmife ; but from the eafy accefs, the little danger of wounding any important artery, and the depending fituation, I fhall not, on any future occafion, hefitate a moment to open a tumour in a fimilar fituation, by puncturing through the vagina, if within reach of the trocar.

We have greatly to lament this as an additional inftance, that even examinations, poft mortem, will not always folve the myfteries we wifh to unfold. Should, however, the experience of any of your Correfpondents fuggeft a folution of thefe difficulties, I fhall be happy to have my mind informed, wherein I confefs it is bewildered. If the cafe was lumbar abfcefs, complicated with an encyfted tumour, why did we not meet with the cyft ? Could it be diffolved ? If merely a lumbar abfcefs, why did not pus follow the trocar ? But it is intricate ; and fhould I learn that it has afforded inftruction on any future emergency, I fhall derive ample fatisfaction for being inftrumental to its publication.

*May* 31, 1802.

